# Complement-dependent bystander injury to neurons in AQP4-IgG seropositive neuromyelitis optica

**DOI:** 10.1186/s12974-018-1333-z

**Published:** 2018-10-22

**Authors:** Tianjiao Duan, Alex J. Smith, Alan S. Verkman

**Affiliations:** 1 Departments of Medicine and Physiology, University of California, 1246 Health Sciences East Tower, 513 Parnassus Ave, San Francisco, CA 94143-0521 USA; 2 Department of Neurology, Second Xiangya Hospital of Central South University, Changsha, 410011 Hunan People’s Republic of China

**Keywords:** NMO, Aquaporin-4, Astrocyte, Neuron, Complement

## Abstract

**Background:**

Aquaporin-4-immunoglobulin G (AQP4-IgG) seropositive neuromyelitis optica spectrum disorder (herein called NMO) is an autoimmune disease of the central nervous system in which AQP4-IgG binding to AQP4 on astrocytes results in complement-dependent astrocyte injury and secondary inflammation, demyelination, and neuron loss. We previously reported evidence for a complement bystander mechanism for early oligodendrocyte injury in NMO. Herein, we tested the hypothesis that complement bystander injury, which involves diffusion to nearby cells of activated soluble complement components from complement-injured astrocytes, is a general phenomenon that may contribute to neuronal injury in NMO.

**Methods:**

Primary cocultures of rat astrocytes and cortical neurons were established to study complement-dependent cell death after exposure to AQP4-IgG and complement. In animal experiments, AQP4-IgG was delivered to adult rats by intracerebral injection. Cell cultures and rat brain were studied by immunofluorescence.

**Results:**

In primary astrocyte-neuron cocultures, addition of AQP4-IgG and complement resulted in death of neurons nearby astrocytes. Deposition of complement membrane attack complex C5b-9 was seen on neurons nearby astrocytes, whereas C1q, the initiating protein in the complement pathway, was seen only on astrocytes. Neuron death was not seen with a complement inhibitor, with C1q- or C6-depleted complement, in pure neuron cultures exposed to AQP4-IgG and complement or in cocultures exposed to an astrocyte toxin. Intracerebral injection in rats of AQP4-IgG and a fixable dead cell fluorescent marker produced death of neurons near astrocytes, with C5b-9 deposition. Neuron death was not seen in rats receiving a complement inhibitor or in AQP4-IgG-injected AQP4 knockout rats.

**Conclusion:**

These results support a novel mechanism for early neuron injury in NMO and provide evidence that complement bystander injury may be a general phenomenon for brain cell injury following AQP4-IgG-targeted astrocyte death.

**Electronic supplementary material:**

The online version of this article (10.1186/s12974-018-1333-z) contains supplementary material, which is available to authorized users.

## Background

Aquaporin-4-immunoglobulin G (AQP4-IgG) seropositive neuromyelitis optica spectrum disorder (herein called NMO) is an autoimmune demyelinating disease of the central nervous system. NMO pathogenesis involves binding of AQP4-IgG autoantibodies to water channel AQP4 on astrocytes, resulting in complement- and cell-mediated astrocyte injury, inflammation, demyelination, and neuron loss [1–3]. Though demyelination and neuronal injury could be secondary consequences of astrocyte death and an inflammatory response, the rapid disease progression seen in some NMO patients [4, 5] and the rapid pathological changes seen in animal models during in vivo imaging of lesion formation [6, 7] suggest more direct mechanisms by which astrocyte injury produces neuronal injury and neurologic deficit. Several mechanisms have been proposed to account for neuronal injury in NMO, including excitotoxic damage following glutamate release from injured astrocytes [8, 9] and secondary recruitment of granulocytes and cytotoxic T cells [10, 11]. However, the role of excitotoxic mechanisms has been controversial [12, 13], and although cellular mechanisms are probably important, they are unlikely to cause the immediate damage to surrounding cells following exposure to AQP4-IgG.

Complement activation is a major effector pathway in NMO. NMO pathology in humans shows centrovascular deposition of activated complement [14–16], and early clinical trials data support the efficacy of a complement inhibitor [17, 18]. Complement-dependent NMO pathology is also seen in experimental animal models of NMO produced by passive transfer of AQP4-IgG [6, 7, 19]. We recently reported evidence for complement bystander injury to oligodendrocytes, in which complement activation following AQP4-IgG binding to AQP4 on astrocytes results in killing of nearby oligodendrocytes by a bystander mechanism involving local diffusion of activated, soluble complement components, leading to formation of the complement membrane attack complex (MAC) on oligodendrocytes [20]. Complement bystander injury has been reported before in Rasmussen’s encephalitis [21], cerebral artery smooth muscle cells [22], and on cells surrounding amyloid plaques in postmortem samples from Alzheimer’s patients [23]. Low expression of CD59, a membrane-anchored complement regulator protein that inhibits MAC formation on target cells [24, 25], appears to be important for complement bystander injury, probably because of the limited transfer of soluble, metastable C5b67 from the primary target cell to nearby bystander cells. Neurons have been reported to express low levels of endogenous complement inhibitors, including CD59 [26–28], and are therefore potential targets for bystander damage.

Here, we tested the hypothesis that complement bystander injury is a general pathogenic mechanism in NMO, accounting not only for early oligodendrocyte injury and demyelination, but also for direct neuronal injury. This study was motivated by in vivo pilot experiments in rat brain in which neurons were identified as being frequently injured, along with oligodendrocytes, soon after intracerebral AQP4-IgG injection. Here, experiments done in astrocyte-neuron cocultures and in rat brain show that AQP4-IgG and complement do not injure neurons directly, but kill neurons in close proximity to astrocytes by a complement bystander mechanism.

## Methods

### Materials

Recombinant purified AQP4-IgG (rAb-53) [29, 30] was provided by Dr. Jeffrey Bennett (University of Colorado, Aurora, CO). Fc hexamer Fc-μTP-L309C was as described [31]. Chemicals were purchased from Sigma-Aldrich (St. Louis, MO) unless specified otherwise. Sprague-Dawley rats were purchased from Charles River Laboratories (Wilmington, MA) and bred at UCSF. AQP4^−/−^ rats for control studies were generated by CRISPR/Cas9 as reported [32]. All animal procedures were approved by the University of California, San Francisco Animal Care and Use Committee (IACUC).

### Cell culture

Primary cortical neuron cultures were generated from the brains of embryonic day 18 (E18) Sprague-Dawley rats (timed-pregnant, Charles River Laboratories, Wilmington, MA), as described [33, 34], with modification. Briefly, the cerebral hemispheres were isolated and cortical tissue was placed in cold Hank’s balanced salt solution (HBSS, pH 7.2; Invitrogen, Camarillo, CA) without Ca^2+^ and Mg^2+^. After removal of the meninges, tissue was diced, incubated for 10 min in 0.25% trypsin-EDTA at 37 °C, and triturated with an 18-gauge needle. The single-cell suspension was passed through a 70-μm nylon strainer (Falcon, Corning, NY) and centrifuged. The tissue pellet was resuspended in Neurobasal medium containing 2% B27-supplement and 0.5 mM Glutamax (Gibco, Grand Island, NY). Cells were plated on PDL-coated 12-well plates at the density of 2 × 10^5^/ml. After 5–7 days in culture, neurons were used for experiments.

Primary astrocyte cultures were generated from cerebral cortex of neonatal wild-type and AQP4^−/−^ rats at day 1 post-birth (P1), as described [35, 36]. Briefly, the cerebral hemispheres were isolated and cortical tissue was minced and incubated for 10 min at 37 °C in 0.25% trypsin-EDTA. Dissociated cells were centrifuged at 500*g* for 5 min and resuspended in Dulbecco’s modified Eagle medium (DMEM) containing 10% FBS and 1% penicillin/streptomycin in T75 flasks. After cell confluence (8–10 days), flasks were shaken in a rotator at 180 rpm overnight to purify astrocytes. The medium was replaced with DMEM containing 3% FBS and 0.25 mM dibutyryl cAMP to induce differentiation. Cultures were maintained for up to an additional 2 weeks. For cocultures, astrocytes were plated on neurons and cocultured in neuron medium overnight before experiments. The neuron:astrocyte ratios of cocultures were from 5:1 to 20:1.

### Complement-dependent cytotoxicity

Specified concentrations of AQP4-IgG (or control human IgG, Thermo Fisher Scientific, Rockford, IL) and human complement (Innovative Research, Novi, MI) were added in Hank’s buffer, and cells were incubated at 37 °C for specified times. In some experiments, cells were exposed to serum of an AQP4-IgG seropositive NMO patient who met the revised diagnostic criteria for clinical disease. A fixable dead-cell stain (amine-reactive dye, Invitrogen, Eugene, OR) at 1:1000 dilution was added 30 min prior to cell fixation. In some experiments, C1q- or C6-deficient human complement (Innovative Research, Novi, MI) was used instead of normal complement. In some experiments, the astrocyte toxin α-aminoadipic acid (Santa Cruz Biotechnology, Dallas, TX) at 2 mM was added to astrocyte-neuron cocultures for 75 min.

For live-cell real-time imaging, astrocyte-neuron cocultures were grown on 6-well plates and imaged by phase-contrast optics using a 20×, 0.45 NA objective lens on a Nikon Eclipse Ti microscope equipped with an environmental chamber at 37 °C and 5% CO_2_. Ethidium homodimer-1 (1 μM, Invitrogen, Eugene, OR) was added to the culture medium prior to image acquisition. Transmitted light (phase-contrast) and red fluorescence images were obtained sequentially every 2 min for a 30-min baseline period and then for 2 h following addition of 20 μg/ml AQP4-IgG and 2% complement.

### Rat studies

AQP4-IgG was delivered to adult wild-type or AQP4^−/−^ rats by intracerebral injection. Rats were anesthetized with ketamine (100 mg/kg) and xylazine (10 mg/kg) and mounted on a stereotaxic frame. Following a midline scalp incision, a 1-mm-diameter burr hole was drilled 0.5 mm anterior and 3.5 mm lateral to the bregma for insertion of a glass pipette with a 40-μm-diameter tip to a depth of 3 mm. AQP4-IgG (or control IgG, each 15 μg) together with 6 μM fixable dead cell dye ethidium homodimer-1 (EH-1) was infused in a volume of 3 μl over 6 min by pressure injection. The glass pipette was kept in place for 10 min before withdrawal to prevent leaking. In some studies, the Fc hexamer Fc-μTP-L309C (50 mg/kg, iv) or MK801 (10 mg/kg, ip) was administered 2 h or 30 min, respectively, before intracerebral injection of AQP4-IgG. At 90 min, rats were deeply anesthetized and transcardiacally perfused with 200 ml heparinized PBS and 200 ml of 4% paraformaldehyde (PFA) in PBS. Brains were removed and post-fixed for 4 h in 4% PFA and cryoprotected in 20% sucrose for cutting 7-μm-thick sections on a cryostat.

### Immunofluorescence

Following treatments, cell cultures were rinsed in PBS, fixed with 4% PFA for 15 min, and then blocked with 1% BSA and 0.2% Triton-X100 in PBS for 1 h. Cultures were incubated at room temperature for 2 h, and brain sections were incubated at 4 °C overnight with antibodies against AQP4 (1:200, Santa Cruz Biotechnology), GFAP (glial fibrillary acidic protein, 1:1000; Millipore), MAP2 (microtubule-associated protein 2, 1:100, Thermo Fisher Scientific), NeuN (Neuronal Nuclei, 1:200; Millipore), C1q (1:50, Abcam, Cambridge, MA), C5b-9 (1:100, Santa Cruz Biotechnology), or CD59 (5 μg/ml, Lifespan Bioscience), followed by the appropriate species-specific Alexa Fluor-conjugated secondary antibody for 1 h (5 μg/ml each, Invitrogen). In some control studies, phosphatidylinositol-specific phospholipase C (PI-PLC) (0.5 U/ml, Invitrogen) was added 1 h prior to experiments to release the extracellular portion of glycophosphoinositol (GPI)-anchored membrane protein CD59. AQP4-IgG was detected using Alexa Fluor-conjugated anti-human IgG. Sections were mounted with VectaShield (Vector Laboratories, Burlingame, CA), and immunofluorescence was visualized on a Nikon confocal microscope using a 20×/0.5 N.A., 60×/1.25 N.A., or 100×/1.4 N.A. oil objective lens.

## Results

### Characterization of astrocyte-neuron cocultures

**Fig. 1:**
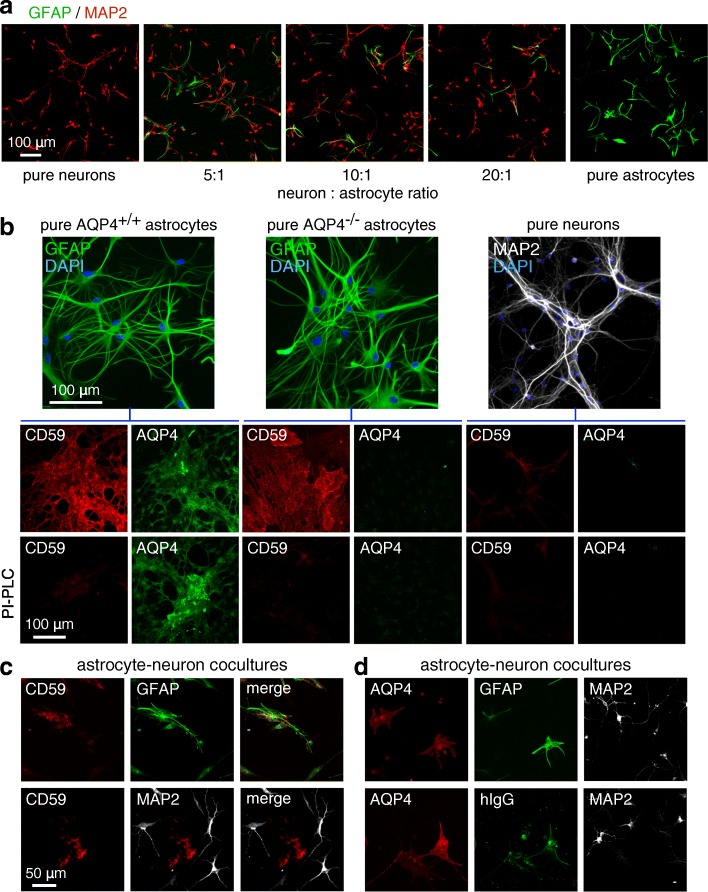
Characterization of rat astrocyte-neuron cocultures. **a** GFAP and MAP2 immunofluorescence of pure neuron cultures, pure astrocyte cultures, and astrocyte-neuron cocultures at indicated cell ratios. **b** (top panels) GFAP or MAP2 immunofluorescence (with DAPI counterstaining) of pure AQP4^+/+^ and AQP4^−/−^ astrocyte cultures and pure neuron cultures. (lower panels) CD59 and AQP4 immunofluorescence. Where indicated (bottom row), cells were incubated with PI-PLC 1 h prior to fixation. **c** CD59 immunofluorescence of astrocyte-neuron cocultures costained with GFAP or MAP2. **d** (upper) AQP4 immunofluorescence of astrocyte-neuron cocultures costained with GFAP and MAP2. (lower) AQP4-IgG (human IgG, hIgG) immunofluorescence (visualized with secondary anti-human IgG antibody) in astrocyte-neuron cocultures following 1 h incubation with 20 μg/ml AQP4-IgG, costained with AQP4 and MAP2. Micrographs representative of studies done of three sets of cultures

An astrocyte-neuron coculture model was established with the goal of having relatively few, well-separated astrocytes with many surrounding neurons in order to facilitate imaging of bystander injury to neurons. Figure 1a shows staining for GFAP (astrocyte marker) and MAP2 (neuron marker) in pure astrocyte and neuron cultures and in cocultures generated using different cell ratios. The astrocyte cultures were fully differentiated using dibutyryl-cAMP for these studies, and astrocyte-neuron cocultures were generated as described under the “Methods” section. A neuron:astrocyte cell ratio of 20:1, which showed relatively few and well-separated astrocytes, was used for subsequent experiments.

Figure 1b (top panels) shows that the individual astrocyte and neuron cultures were > 95% pure as seen by GFAP and MAP2 immunofluorescence with DAPI costaining. AQP4 and CD59 immunofluorescence showed, as expected, AQP4 expression only on astrocytes, with AQP4-deficient astrocytes generated from AQP4^−/−^ rats as control (Fig. 1b, lower panels). CD59 was expressed on astrocytes, with little expression seen on neurons. The control for CD59 immunofluorescence was treatment with the enzyme PI-PLC, which cleaves the extracellular CD59 antigen from its membrane-spanning domain. In astrocyte-neuron cocultures, CD59 expression was seen on astrocytes and not on neurons (Fig. 1c). At 1 h following incubation with AQP4-IgG, AQP4-IgG was seen on astrocytes but not neurons as detected using a fluorescent anti-human IgG secondary antibody (Fig. 1d). Therefore, astrocytes but not neurons are the target of the pathogenic NMO anti-AQP4 autoantibody.

### Complement bystander killing of neurons in astrocyte-neuron cocultures

**Fig. 2:**
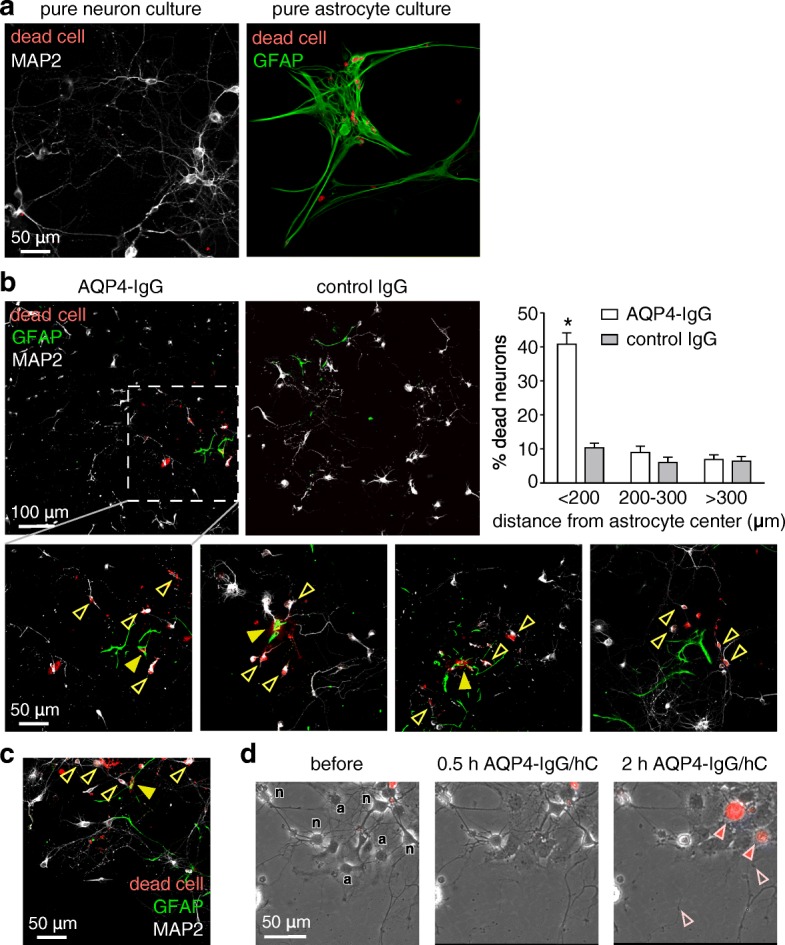
Complement-mediated neuron injury in astrocyte-neuron cocultures. **a** Complement-dependent cytotoxicity in pure neuron and pure astrocyte cultures following incubation with 20 μg/ml AQP4-IgG and 2% human complement for 2 h, with fixable dead cell marker (reactive amine dye, labeled “dead cell”). Cultures were immunostained for GFAP and MAP2, with dead cells stained red. **b** Cocultures were incubated as in **a** and immunostained for GFAP (green) and MAP2 (gray), with dead cells red. Expanded images (lower panels) showing dead astrocytes (filled yellow arrowheads) and nearby dead neurons (open yellow arrowheads) in representative fields. Bar graph at the right shows percentage of dead neurons at different distances from the center of dead astrocytes (mean ± S.E.M., *n* = 6 cultures, total 32 astrocytes imaged, *P < 0.01 comparing AQP4-IgG vs. control IgG). **c** Cocultures were incubated with 1% NMO patient serum and 5% human complement and immunostained as in panel **b**. **d** Panels from time-lapse image sequence (see Additional file 1: Movie 1) showing astrocytes and neurons in coculture before and at 0.5 and 2 h after addition of 20 μg/ml AQP4-IgG and 2% human complement containing dead cell marker ethidium homodimer-1. Labels in the left panel: n, neuron; a, astrocyte. Filled arrowheads point to early damage of neurons in contact with astrocytes, and red color is due to uptake of dead cell marker. Open arrowheads indicate sites of neurite blebbing and degeneration

Complement-dependent cytotoxicity was produced by incubation of astrocyte-neuron cocultures with AQP4-IgG and human complement. A fixable dead cell stain was included in order to visualize dead cells. In pure astrocyte and neuron cultures, > 60% of astrocytes were stained with dead cell dye following 2 h exposure to AQP4-IgG and complement, whereas < 5% of neurons were stained (Fig. 2a). In the astrocyte-neuron cocultures, dead cell-stained astrocytes were seen (Fig. 2b, yellow filled arrowheads), as well as dead cell-stained neurons (yellow open arrowheads), generally in close proximity to astrocytes. Dead cell-stained neurons were seen near both dead cell-stained and non-stained astrocytes. Figure 2b (right) summarizes the percentage of dead neurons at different distances from the center of dead astrocytes, showing preferential killing of neurons within 200 μm of dead astrocytes. A similar pattern of dead neurons nearby astrocytes was seen using NMO patient serum instead of the recombinant AQP4-IgG antibody (Fig. 2c).

Time-lapse imaging was done to visualize in real time the injury to astrocytes and neurons following exposure to AQP4-IgG and complement. Phase-contrast and fluorescence imaging was done to visualize all cells and their injury. Neurons and astrocytes were readily differentiated based on morphological criteria and the weak phase-halo of the very flat astrocytes (Fig. 2d, left panel). Upon addition of AQP4-IgG and complement, astrocytes responded with morphological rearrangements; however, only a subset of cells was damaged severely enough for full membrane permeabilization to occur (not shown). Neurons adjacent to astrocytes showed neurite blebbing and membrane lysis with uptake of ethidium homodimer (Fig. 2d, right panel and Additional file 1: Movie 1).

**Fig. 3:**
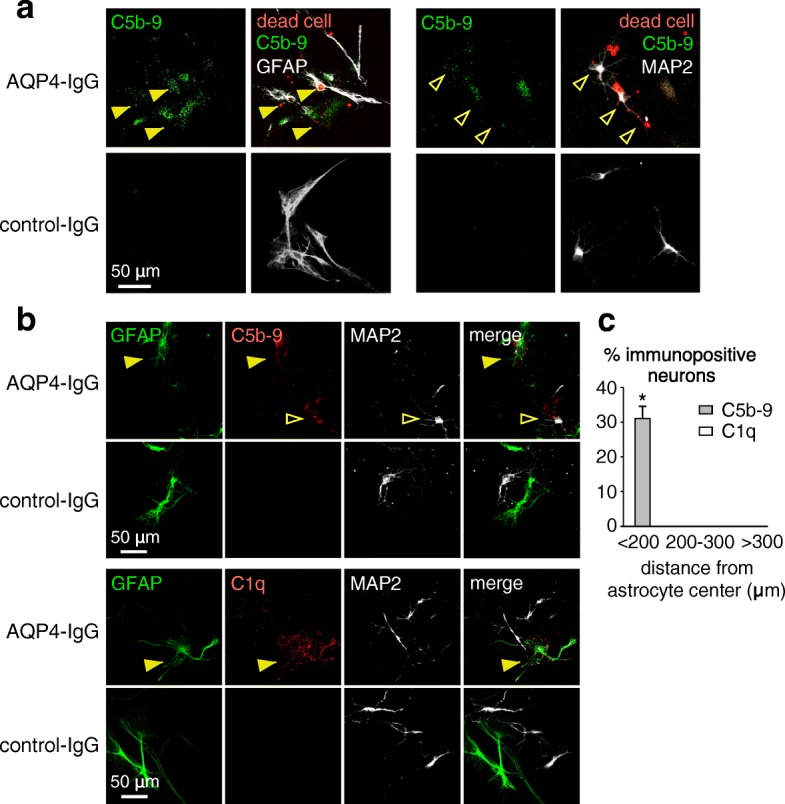
Evidence for a complement bystander mechanism for AQP4-IgG/complement-induced neuron injury in astrocyte-neuron cocultures. **a** C5b-9 immunofluorescence (green) in astrocyte-neuron cocultures with dead cell stain (red) at 2 h after incubation with 20 μg/ml AQP4-IgG and 2% human complement, immunostained for GFAP or MAP2 (gray). **b** C5b-9 and C1q immunofluorescence of cocultures with cell markers GFAP and MAP2 treated as in **a**. Filled arrowheads indicate C5b-9 or C1q on astrocytes, and open arrowheads show C5b-9 on neurons. **c** Percentage of C5b-9 and C1q positive neurons at different distances from C5b-9 or C1q positive astrocytes (mean ± S.E.M., *n* = 6, *P < 0.01 comparing AQP4-IgG vs. control IgG)

C5b-9 and C1q immunostaining was done to investigate whether neuronal injury was caused by a complement bystander mechanism, which would predict C5b-9 on both astrocytes and nearby injured neurons, whereas C1q, the initiating complement protein, only on astrocytes. C5b-9 immunofluorescence was seen on many dead cell-stained astrocytes and neurons in the cocultures (Fig. 3a). Figure 3b indicates C5b-9 deposition on astrocytes as well as on neurons near astrocytes, whereas C1q immunofluorescence was seen only on astrocytes. Figure 3c summarizes the percentage of C5b-9 and C1q positive neurons at different distances from C5b-9 and C1q positive astrocytes. These results support a complement bystander mechanism in which C1q binding to AQP4-IgG on astrocytes results in deposition of the cytotoxic C5b-9 complex on both astrocytes and nearby neurons.

**Fig. 4:**
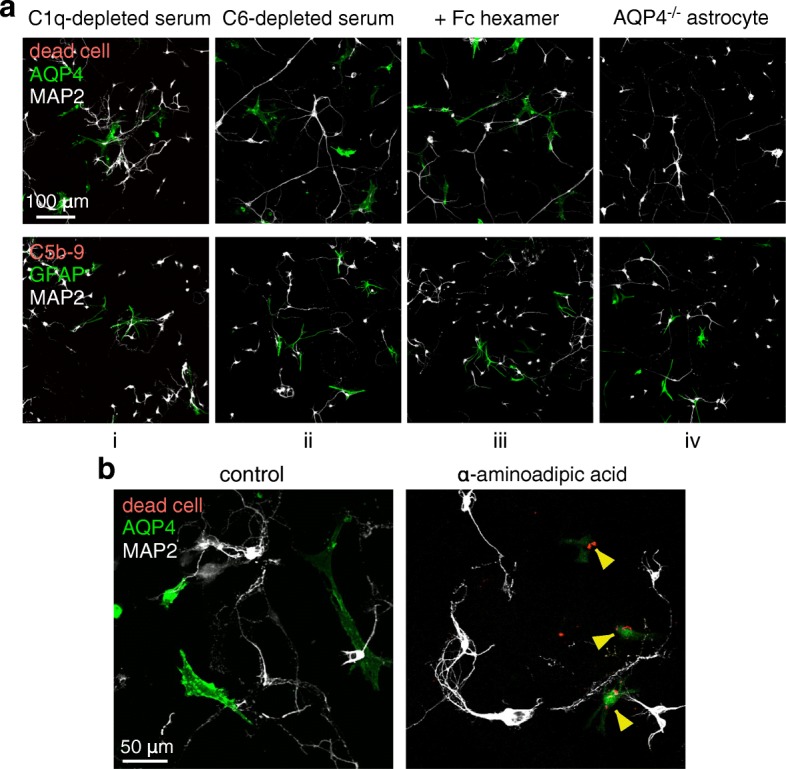
Control studies supporting a complement bystander mechanism for neuron injury. **a** Astrocyte-neuron cocultures were incubated for 2 h with 20 μg/ml AQP4-IgG and 2% C1q-depleted serum (i) or with 20 μg/ml AQP4-IgG and 2% C6-depleted serum (ii); cocultures were incubated for 2 h with 20 μg/ml AQP4-IgG and 2% human complement that was pre-exposed for 1 h to 1 μg/ml Fc hexamer (complement inhibitor) (iii); AQP4^−/−^ astrocyte-neuron cocultures were incubated for 2 h with 20 μg/ml AQP4-IgG and 2% human complement (iv). GFAP, MAP2, and C5b-9 immunofluorescence as indicated, with dead cells stained red. **b** Direct astrocyte injury caused by α-aminoadipic acid. Cocultures were exposed to 2 mM α-aminoadipic acid for 75 min, with AQP4 and MAP2 immunofluorescence and dead cell stain shown. Filled arrowheads show dead astrocytes. Micrographs representative of three sets of studies done on different cocultures

Control studies were done to support a complement bystander mechanism for neuron killing in the astrocyte-neuron cocultures. Cocultures incubated with AQP4-IgG and C1q-depleted serum did not show cell killing or C5b-9 deposition, indicating that activation of classic complement pathway is the effector of bystander injury (Fig. 4a (i)). Incubation of cocultures with AQP4-IgG and C6-depleted serum did not produce cytotoxicity, indicating that neuron killing is the consequence of MAC (C5b-9) deposition rather than upstream anaphylotoxins or other mediators (Fig. 4a (ii)). Cytotoxicity was not seen in the presence of a complement inhibitor (Fig. 4a (iii)) or in AQP4-deficient cocultures in which astrocytes were cultured from AQP4^−/−^ rats (Fig. 4a (iv)). In a separate control, cocultures were incubated with the astrocyte-selective toxin α-aminoadipic acid [37, 38] in which the incubation time and toxin concentration were chosen in initial studies to cause killing of many astrocytes but few neurons (in pure cultures). Exposure of pure cultures to 2 mM α-aminoadipic acid for 75 min resulted in ~ 85% astrocyte death and ~ 10% neuron death. Incubation of cocultures with α-aminoadipic acid under these conditions resulted in killing of astrocytes but not neurons (Fig. 4b), suggesting that mediators or other factors released from dying astrocytes are not responsible for neuronal injury.

### Complement bystander injury to neurons in a rat model of NMO

**Fig. 5:**
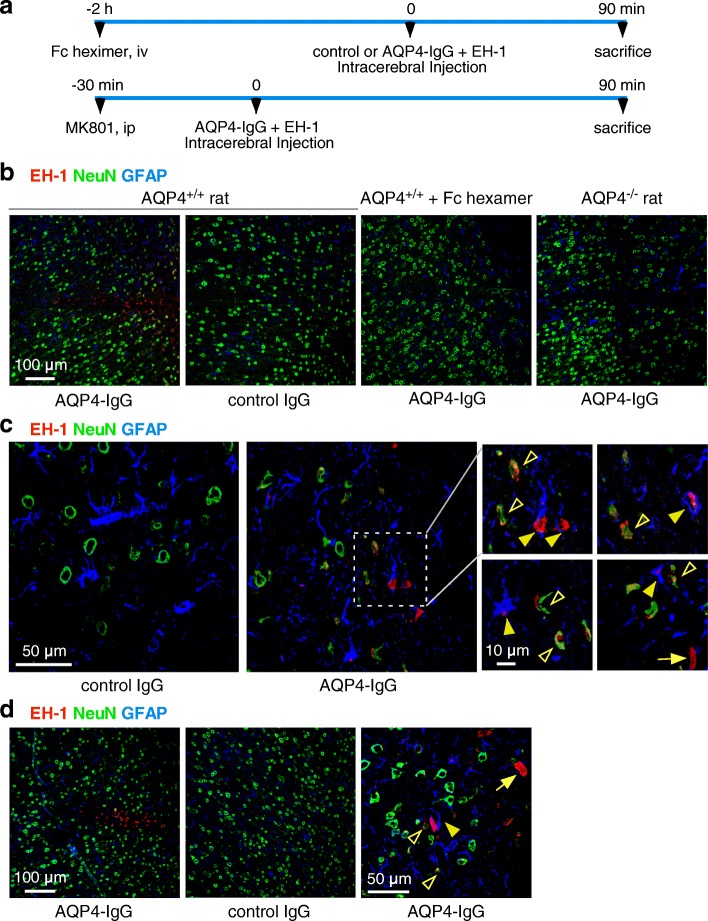
Complement bystander killing of neurons near astrocytes in rat brain following intracerebral AQP4-IgG injection. **a** AQP4-IgG (15 μg) (or 15 μg control IgG) and dead cell stain EH-1 (6 μM) in a 3-μl volume was injected in cortex and striatum of rat brain, and rats were sacrificed at 90 min. In some studies, rats were injected with Fc hexamer (50 mg/kg, iv) by tail vein 2 h before or MK801 (10 mg/kg, ip) 30 min before intracerebral injection of AQP4-IgG. **b** Low-magnification micrographs showing dead cells (red EH-1 fluorescence), NeuN (green), and GFAP (blue) for studies done in AQP4^+/+^ rats, Fc hexamer-treated AQP4^+/+^ rats, and AQP4^−/−^ rats. **c** High-magnification confocal images of AQP4^+/+^ rat brain at 90 min after injection of AQP4-IgG (or control IgG) and EH-1 showing dead astrocytes and nearby dead injured neurons. Expanded images on the right showing representative fields. Filled arrowheads show dead astrocytes, open arrowheads show nearby dead neurons, and arrow points to a non-neuron, non-astrocyte dead cell. **d** Rats were injected with MK801 (10 mg/kg, ip) 30 min before intracerebral injection of AQP4-IgG (or control IgG) and EH-1. Imaging showing dead cells (red EH-1 fluorescence), NeuN (green), and GFAP (blue). Filled arrowheads show dead astrocytes, open arrowheads show nearby dead neurons, and arrow points to a non-neuron, non-astrocyte dead cell. Representative of micrographs done on sections from three rats

To investigate complement bystander injury in vivo, rats were administered AQP4-IgG by intracerebral injection, together with the dead cell dye ethidium homodimer-1 (EH-1) (Fig. 5a). Rats were sacrificed at 90 min, and brains were perfusion-fixed for frozen sections. Figure 5b shows many dead cells near the needle tract in rats receiving AQP4-IgG. In control studies, few or no dead cells were seen with injection of non-NMO human IgG instead of AQP4-IgG, when rats were pre-treated with the Fc hexamer complement inhibitor or when AQP4-IgG was injected in AQP4^−/−^ rats. NeuN immunofluorescence of neurons with GFAP immunofluorescence of astrocytes showed many red-stained dead astrocytes, as well as nearby dead neurons and some other cell types not stained with NeuN or GFAP (Fig. 5c). In the area within and just around the needle tract, ~ 80% of astrocytes were dead (red-stained), with ~ 55% of dead neurons nearby (within 200 μm) astrocytes (sections from 3 rats examined).

To investigate if excitotoxic mechanisms might contribute to the early neuron injury observed in response to AQP4-IgG, the NMDA receptor antagonist MK801 (10 mg/kg, ip) [39, 40] was injected 30 min before intracerebral injection of AQP4-IgG and EH-1. Figure 5d shows similar EH-1 staining by neurons in the presence and absence of MK801, suggesting minimal contribution of excitotoxicity to neuron cell death in this model.

**Fig. 6:**
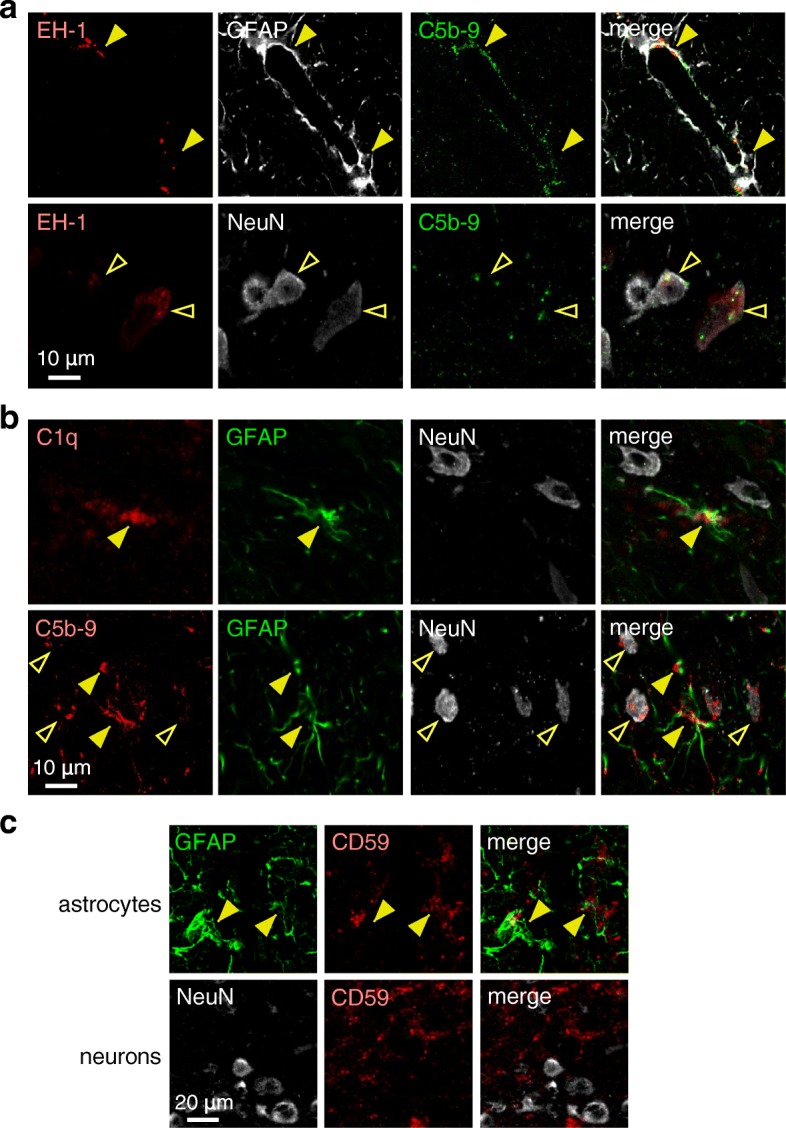
Evidence for complement bystander killing of neurons in rat brain. **a** Brains were injected with AQP4-IgG and EH-1 and harvested at 90 min as in Fig. 5. High-magnification confocal micrographs showing colocalization of EH-1, GFAP, and C5b-9 (top, filled arrowheads) and EH-1, NeuN, and C5b-9 (bottom, open arrowheads). **b** Brains were injected with AQP4-IgG alone (without EH-1) and harvested at 90 min. C1q colocalization with GFAP and NeuN, and C5b-9 colocalization with GFAP and NeuN (bottom). Filled arrowheads show C5b-9 or C1q deposition on astrocytes, and open arrowheads show C5b-9 on neurons. **c** CD59 immunofluorescence with GFAP or NeuN in control (non-injected) rat. Filled arrowheads indicate CD59 on astrocytes. Representative of micrographs done on sections from three rats

C5b-9 and C1q immunofluorescence in rats injected with AQP4-IgG and EH-1 showed C5b-9 on dead astrocytes as well as on nearby dead neurons (Fig. 6a). In rats injected with AQP4-IgG (without EH-1), immunofluorescence of C1q, GFAP, and NeuN showed C1q deposition only on astrocytes (Fig. 6b, top panels), whereas C5b-9 was seen on both astrocytes and nearby neurons (Fig. 6b, bottom panels). Figure 6c shows CD59 colocalization with GFAP in astrocytes, but not with NeuN on neurons in (non-injected) rat brain, which is consistent with the cell culture studies showing CD59 on astrocytes but not neurons.

## Discussion

This study reports evidence for complement bystander killing of neurons in astrocyte-neuron cocultures in vitro and in rats in vivo. Astrocyte-neuron cocultures exposed to AQP4-IgG or NMO patient serum and complement showed injury and death in neurons very near astrocytes. Time-lapse imaging revealed early injury to neurons near astrocytes even before gross plasma membrane permeabilization and uptake of the dead cell stain. Neuron death was not seen in the absence of AQP4-IgG or with complement inhibition, with C1q- or C6-depleted serum, or with an astrocyte toxin, implicating a complement-dependent mechanism initiated by AQP4-IgG binding to astrocytes. The deposition of C5b-9 on astrocytes and injured nearby neurons, with C1q deposition only on astrocytes, indicates that activation of the classical complement cascade on astrocytes leads to MAC deposition on both astrocytes and nearby neurons. Astrocyte killing by a selective toxin in the cocultures did not result in neuron killing, indicating the factors released from dead astrocytes are not responsible for neuron killing in the coculture experiments. In rats in vivo, dead neurons near dead astrocytes were seen at 90 min after intracerebral administration of AQP4-IgG, with C5b-9 deposition on astrocytes and neurons, but C1q deposition only on astrocytes. Neuron cytotoxicity was not seen with complement inhibition, with non-NMO human IgG, or in AQP4^−/−^ rats. Though it is not possible to exclude mechanisms other than complement bystander injury to explain the early neuron death following AQP4-IgG in vivo, similar levels of neuronal damage were observed in control rats and rats treated with the NMDA receptor antagonist MK801, suggesting that excitotoxic mechanisms do not substantially contribute to the observed cell death.

The generality of complement bystander injury in NMO adds to the list of proposed mechanisms linking the AQP4-IgG astrocytopathy to downstream neurological deficit and has potential implications for treatment of NMO. Complement bystander injury to neurons could account for the early and marked neurological deficit seen in some NMO patients, as well as for the rapid early neuron loss in experimental animal models of NMO. In addition to neuronal injury from a complement bystander mechanism, as reported here, and oligodendrocyte injury as we reported before [20], we speculate that other cell types in the central nervous system may be injured similarly, such as microvascular endothelial cells and pericytes lining the blood-brain barrier in close contact with AQP4-enriched astrocyte foot processes. With regard to NMO therapeutics, early loss of neuron and axons would limit the potential efficacy of remyelination therapeutics [41, 42]. Complement bystander injury would be prevented by inhibition of the classical complement pathway or earlier steps in NMO pathogenesis such as AQP4-IgG binding to AQP4 or by drugs or maneuvers to increase CD59 expression in the secondarily injured cells. Complement bystander injury would be relatively insensitive to drugs acting on more downstream disease pathogenesis mechanisms such as general immunosuppressants.

Though the data here provide strong evidence for complement bystander killing of neurons, several limitations are noted. As human specimens were not studied, the significance of complement bystander injury to neurons in human NMO is uncertain. Even if specimens from humans with active NMO disease were available, it would likely be difficult to identify dead neurons because of their clearance, and the lack of a dead cell stain and defined AQP4-IgG exposure time as in the rat studies here. Though the in vitro cocultures allowed clear-cut interpretation of the immunofluorescence data because of the well-demarcated cell distribution in two dimensions and the specification of precise solution composition, the culture system does not recapitulate many aspects of the central nervous system, such as the blood-brain barrier, the complex three-dimensional network of many different brain cell types, and inflammatory effectors. It is therefore not possible in the in vivo rat studies to exclude contributions from additional mechanisms of early neuronal injury following astrocyte death, such as excitotoxic injury or injury caused by inflammatory mechanisms such as cytokine release by astrocytes or microglial activation.

## Conclusions

In conclusion, the evidence here for a complement bystander mechanism for neuronal injury in NMO supports the generality of complement bystander injury to brain cell types implicated in NMO pathology, including neurons, oligodendrocytes, and perhaps microvascular endothelia and other cell types. Bystander injury may also be relevant to cellular injury mechanisms such as leukocyte degranulation following AQP4-IgG-induced antibody-dependent cellular cytotoxicity.

## Additional file

Additional file 1: Movie 1. Time-lapse imaging of astrocyte-neuron coculture following addition of AQP4-IgG and complement as in Fig. 2. Cultures were imaged for 30 min before and 2 h following addition of AQP4-IgG and complement. (AVI 4660 kb)

## Abbreviations

AQP4: Aquaporin-4
AQP4-IgG: Aquaporin-4-immunoglobulin G
DMEM: Dulbecco’s modified Eagle medium
EH-1: Ethidium homodimer-1
GFAP: Glial fibrillary acidic protein
HBSS: Hank’s balanced salt solution
hIgG: Non-NMO pooled human IgG
MAP2: Microtubule-associated protein 2
NeuN: Neuronal Nuclei
NMO: Neuromyelitis optica
PFA: Paraformaldehyde
PI-PLC: Phosphatidylinositol-specific phospholipase C
